# Downregulation of ROCK2 through Nanocomplex Sensitizes the Cytotoxic Effect of Temozolomide in U251 Glioma Cells

**DOI:** 10.1371/journal.pone.0092050

**Published:** 2014-03-18

**Authors:** Xiaojun Wen, Amin Huang, Zhonglin Liu, Yunyun Liu, Jingyang Hu, Jun Liu, Xintao Shuai

**Affiliations:** 1 Department of Neurology, Sun Yat-sen Memorial Hospital,Sun Yat-sen University, Guangzhou, China; 2 State Key Laboratory of Oncology in Southern China, Sun Yat-sen University Cancer Center, Guangzhou, China; 3 Department of Neurology, the Sixth Affiliated Hospital, Sun Yat-sen University, Guangzhou, China; 4 Center of Biomedical Engineering, Zhongshan School of Medicine, Sun Yat-sen University, Guangzhou, China; Sun Yat-sen University Cancer Center, China

## Abstract

**Objective:**

Rho-associated coiled-coil kinase 2 (ROCK2) is an attractive therapeutic target because it is overexpressed in many malignancies, including glioma. Therefore, we designed the current study to determine whether the downregulation of ROCK2 would sensitize the cytotoxic effect of temozolomide (TMZ) in U251 cells.

**Methods:**

Glycol-polyethyleneimine (PEG-PEI) was used to deliver siROCK2 to U251 cells, and the physical characteristics of the PEG-PEI/siROCK2 complex (referred to as the siROCK2 complex) were investigated. The transfection efficiency and cell uptake were determined by flow cytometry (FCM) and confocal laser microscopy (CLSM), respectively. U251 cells were then treated with 100 μM TMZ, siROCK2 complexes or their combination. The apoptosis rate and cell migration were measured by FCM and wound-healing assay, respectively. The levels of Bax, Bcl-2, cleaved caspase-3, MMP-2, and MMP-9 were detected to analyze the degrees of apoptosis and migration.

**Results:**

Our results revealed that the characteristics of the siROCK2 complexes depended closely on the N/P ratios. PEG-PEI served as a good vector for siROCK2 and exhibited low cytotoxicity toward U251 cells. The CLSM assay showed that the siROCK2 complexes were successfully uptaken and that both the protein and mRNA levels of ROCK2 were significantly suppressed. Furthermore, the combination treatment induced a higher apoptosis rate and markedly increased the gap distance of U251 cells in the wound-healing assay. Levels of the proapoptotic proteins Bax and cleaved caspase-3 were significantly increased, whereas levels of the antiapoptotic protein Bcl-2 and the migration-related proteins MMP-2 and MMP-9 were significantly reduced by the combination treatment compared with either treatment alone.

**Conclusions:**

In conclusion, our results demonstrate that the combination of TMZ and siROCK2 effectively induces apoptosis and inhibits the migration of U251 cells. Therefore, the combination of TMZ and siROCK2 complex is a potential therapeutic approach for human glioma.

## Introduction

Gliomas, which are characterized by rapid cell proliferation, high invasion and migration properties, are the most common malignant tumors in the CNS [1]. After standard therapeutics, including surgery, radiation and chemotherapy, the median survival time of glioma patients is approximately 15 months [2]. Despite recent advances in all of these therapies, the response is still very unfavorable. One of the major factors contributing to the failures of these therapies is the highly infiltrative nature of the glioma toward adjacent normal brain tissue.

RhoA, one member of the small GTPases, is involved in a number of cellular events, including cell cycle progression, differentiation, cell invasion and migration [3]. ROCK2 is one of the RhoA effectors that were determined to be players in RhoA-mediated actin-myosin contractility and focal adhesion assembly [4]. The overexpression of RhoA/ROCK has been reported in many tumor types, including glioma [5], [6]. Studies have shown that the Rho/ROCK signal is overexpressed in glioblastoma (GBM) cells and correlates positively with the degree of malignancy in astrocytoma [6]–[8]. In addition, a high expression of RhoA induces metastatic properties and increases tumor invasiveness [5]. Previous studies have also demonstrated that Fasudil, a ROCK2 inhibitor, can suppress the proliferation, migration, invasiveness, and the induction of apoptosis in GBM cells [5], [9].

Based on its high specificity to the target mRNA, RNA interference is considered a promising strategy for the selective downregulation of pathologically overexpressed genes. Non-viral vectors, such as PEI, have been considered an effective approach for siRNA delivery [10], [11]. PEI was chemically modified by the addition of PEG to enhance the biocompatibility and reduce the toxicity of PEI-based complexes [12].

It is well known that gliomas are relatively resistant to the chemotherapeutic drug TMZ. Cancer invasion and drug-resistance are increasingly being recognized as interconnected processes that promote disease progression and therapy failure [13]. However, the effects and mechanisms underlying ROCK2 silencing by the siROCK2 complex in U251 cells, which sensitizes the chemotherapeutic effects of TMZ, have not been studied. Thus, we hypothesized that siROCK2 can be taken into consideration in the administration of the TMZ chemotherapeutic. In the present study, we demonstrated that the siROCK2 complexes can efficiently transfect U251 cells and show inhibitory effects on apoptosis via the blockage of ROCK2. Furthermore, we showed that the downregulation of ROCK2 and TMZ has a synergistic effect on apoptosis and cell migration.

## Materials and Methods

### Materials

siROCK2, negative control siRNA (siNC), and Cy3-labeled siROCK2 were designed and synthesized by GenePharma (Shanghai, China). TMZ was purchased from Sigma-Aldrich (St. Louis, MO, USA). Rabbit anti-human antibodies against ROCK2, Bax, Bcl-2, cleaved-caspase-3, and GAPDH and the secondary antibody (goat anti-rabbit) were purchased from Cell Signaling Technology (Beverly, MA, USA).

### Cell culture

The human glioma cell line U251 was purchased from the Chinese Academy of Sciences Cell Bank. The cells were maintained in Dulbecco's modified Eagle's medium (DMEM) supplemented with 10% fetal bovine serum and 1% penicillin-streptomycin at 37°C in an incubator with 5% CO_2_.

### Physical characteristics of the siROCK2 complex

The synthesis of the PEG-PEI copolymers and the physical characteristics of the siROCK2 complexes were described in our previous publication [10]. The PEG-PEI copolymers were synthesized in the Center of Biomedical Engineering, Zhongshan School of Medicine, Sun Yat-sen University. PEG (2 k)-PEI (25 k) was characterized through ^1^H NMR in deuterium oxide. The major resonance peaks of the copolymer in the ^1^H NMR spectrum agree well with the expected chemical structure.

The N/P ratio of the siROCK2 complex denotes the charge ratio between the amino groups of the PEG-PEI and the phosphate groups of the siRNA. This ratio is an important parameter of the nanoparticle. An enhancement of the N/P ratio results in increases in the particle size and decreases in the zeta potential. The cellular uptake of nanoparticles increased with increasing N/P ratio, but the cytotoxicity also increased.

### Gel retardation assay

The condensation of the ROCK2 complex was determined using gel electrophoresis. Briefly, PEG-ROCK2 complexes were formed at various N/P ratios (0, 1, 2, 5, 10, 20, 30, and 40), mixed with 10× loading buffer, and then loaded onto a 1% agarose gel that contained 0.5 μg/mL EB. The electrophoresis parameters were 90 V for 30 min at room temperature. A UV imaging system (Uvidoc, UVItec Ltd., Cambridge, UK) was used to visualize the bands of the ROCK2 complexes.

### Cell viability assay

U251 cells were seeded in 96-well plates at a density of 5000 cells/well in 100 μL of medium and incubated overnight. Various volumes of the PEG-PEI (5 mg/mL) or the siROCK2 complexes (according to different N/P ratios) were added, and the mixtures were incubated for 24 h. The MTT assay was applied to detect the cell viability.

### Transfection efficiency assay

U251 cells were seeded in a six-well plate at a density of 2×10^4^ cells/well. A series of siROCK2 complexes was formed by mixing 80 pmol of Cy3-labeled siRNA and the corresponding amounts of PEG-PEI. After incubation with the siROCK2 complexes for 6 h at 37°C, the medium was removed, and normal culture medium was added. The U251 cells were then incubated in the fresh medium for 18 h. The Lipo2000/siROCK2 (2 μL of Lipo2000 and 80 pmol of siRNA) served as a positive control. The transfection efficiency was determined by FCM.

### Confocal laser microscopy assay (CLSM)

U251 cells were seeded into a confocal laser dish for 24 h and then incubated with Cy3-labeled-siROCK2 complexes at an N/P ratio of 50. After 6 h, the cells were washed three times with PBS. The DNA-staining agent Hoechst 33342 (1 mg/mL) was then added, and the cells were stained for 10 min. The cells were then washed three times with PBS and visualized using a Zeiss LSM 510 META microscope (Carl Zeiss Co., Ltd., Gottingen, Germany).

### Quantitative real-time PCR (RT-PCR)

U251 cells were seeded onto six-well plates and divided into five groups: the blank control group, the siNC group (N/P  = 50), the siROCK2 group (N/P  = 50), the TMZ (100 μM) group and the TMZ (100 μM) + siROCK2 (N/P  = 50) group. The concentration of TMZ was 100 μM, which is in line with a previous publication [14]. After treating for 24 h, the total RNA was extracted using the Trizol reagent according to the manufacturer's instructions. The sequences of the primers are shown in Table 1. Reverse transcription and RT-PCR were performed in accordance with the protocol recommended by the manufacturers of the SYBR RT-PCR Kit (Takara Shuzo, Shiga, Japan). The relative expression of mRNA was assessed by the comparative ^ΔΔ^Ct method. GAPDH was used as an internal standard.

**Table 1:** Primer sequences used for the real-time quantitative reverse transcription-PCR assays.

Gene	Accession number	Forward primer	Reverse primer
**Bcl-2**	NM_000633	CGGTGGGGTCATGTGTGTG	CGGTTCAGGTACTCAGTCATCC
**Bax**	NM_138764	CCAAGGTGCCGGAACTGA	CCCGGAGGAAGTCCAATGT
**MMP-2**	NM_004530	CTTCCAAGTCTGGAGCGATGT	TACCGTCAAAGGGGTATCCAT
**MMP-9**	NM_004994	GGGACGCAGACATCGTCATC	TCGTCATCGTCGAAATGGGC
**Rock2**	NM_004850	GAGAAATGGGGTGGAAGAAATC	TGCTGTCTATGTCACTGCTGAG
**GAPDH**	NM_002046	GGAGCGAGATCCCTCCAAAAT	GGCTGTTGTCATACTTCTCATGG

### Western blotting assay

U251 cells were seeded onto six-well plates and divided into five groups as described above. After treatment for 48 h, the total protein was extracted using RIPA. Equal amounts of protein were separated by SDS-PAGE and transferred to polyvinylidene difluoride (PVDF) membranes. The membranes were incubated at room temperature for 1 h in blocking buffer (5% nonfat milk, Tris-buffered saline, and Tween-20), incubated with rabbit monoclonal anti-ROCK2, Bcl-2, cleaved-caspase-3, Bax, and GAPDH antibodies (Cell Signaling Technology, USA), and then incubated with anti-rabbit IgG-horseradish-peroxidase (1∶8000, Jackson, MI, USA) for 1 h at room temperature. After exposure to X-ray film, the protein bands were imaged using a UV imaging system. The expression of the housekeeping gene, GAPDH, was used as the control.

### Apoptosis assays

U251 cells were seeded onto six-well plates and divided into five groups as described above. The degree of apoptosis was analyzed 48 h after each treatment. Annexin-V-FITC and propidium iodide (PI) double staining was used to evaluate the percentage of apoptotic cells. Briefly, after treatment, 150,000 cells were trypsinized and centrifuged at 1,000 rpm for 5 min three times and then resuspended in 500 μL of binding buffer. The cells were stained with 5 μL of Annexin-V-FITC and 5 μL of PI and incubated at RT in the dark. The samples were analyzed using a flow cytometer (BD Biosciences, San Jose, CA, USA). Each test was repeated in triplicate.

### Wound-healing assays

For the wound-healing assays, U251 cells were plated in six-well plates at a density of 4×10^5^ cells/well and incubated overnight. The media was then aspirated, and the cells were scratched with a sterile tip. Three representative images were collected. After treatment for 12 h, images of the same regions were collected, and the cell motility ratio for each experimental condition was quantified. The tests were repeated in triplicate.

### Statistical analysis

All of the data are expressed as the means ± standard deviation (SD). The statistical analyses were performed using the SPSS version 13.0 software (SPSS Inc., Chicago, IL, USA). Multiple groups were compared using analysis of variance (ANOVA) followed by post-hoc Fisher's least significant difference (LSD) testing when appropriate. The ImageJ software (Wayne Rasband, NIH, USA) was used to quantify the gap distances in the wound-healing assay and for the quantification of the Western blot bands. A p-value of less than 0.05 was considered statistically significant.

## Results

### Agarose gel retardation assay

**Figure 1:**
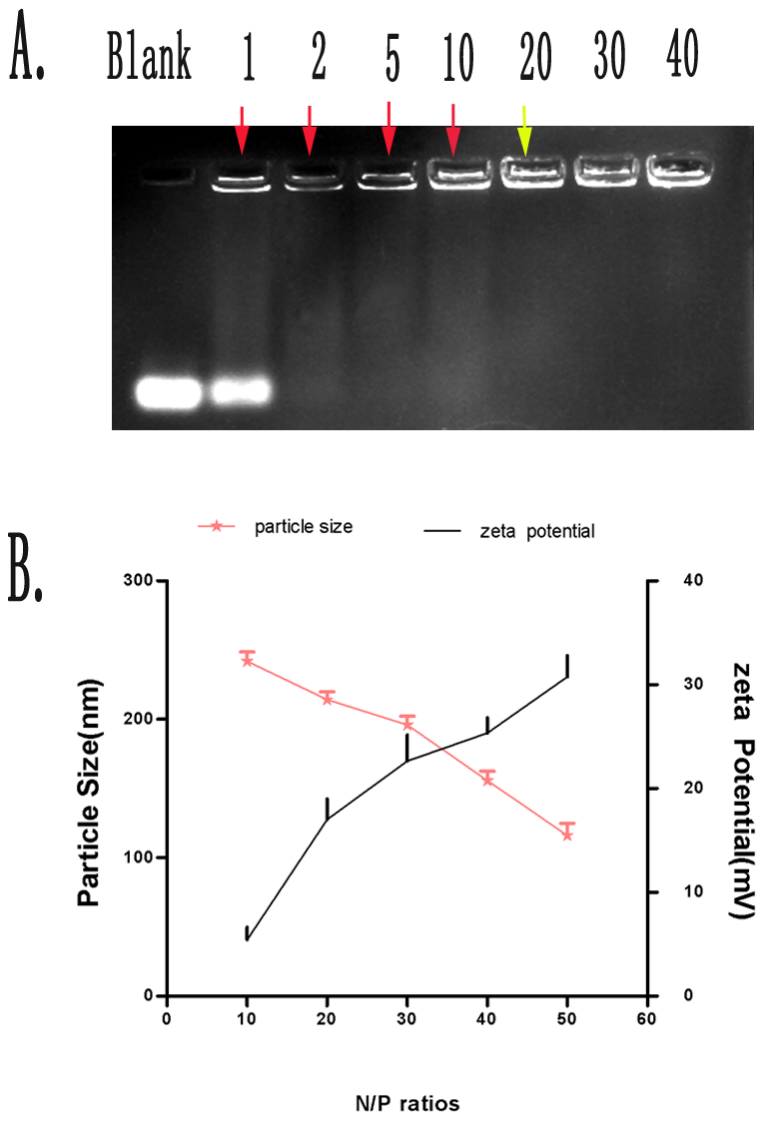
Physical characteristics of siROCK2 complexes. **A** Investigation of siROCK2 condensation by PEG-PEI through gel retardation assays. SiROCK2 complexes were formed at various N/P ratios (blank, 1, 2, 5, 10, 20, 30, and 40); the concentration of siROCK2 was 100 nM. The red arrows indicate partial condensation of SiROCK2 complexes, whereas the yellow arrow indicates complete condensation of SiROCK2 complexes. **B** Particle size and zeta potential of siROCK2 complexes formed at different N/P ratios (10, 20, 30, 40, 50, and 70). The data are shown as the means ± SD; n = 3.

The efficiency of the prepared PEG-PEI to form complexes with siROCK2 molecules via electrostatic interactions was determined through agarose gel retardation (Fig. 1A). We found that the intensity of the siROCK2 bands decreased with increasing N/P ratios (from 0 to 40). The full condensation of siROCK2 was achieved at an N/P ratio of approximately 20, which lead to the disappearance of the siROCK2 band on the agarose gel.

### Particle size and zeta potential of siROCK2 complexes

The appropriate particle size and zeta potential of siROCK2 complexes is essential for its uptake by cells. As shown in Fig. 1B, an increase in the N/P ratio led to a decrease in the size of the siROCK2 complexes and an increase in the zeta potential. At an N/P ratio of 10, the average particle size was 242.10±6.5 nm, and the zeta potential was 5.4±1.2 mV. In contrast, at an N/P ratio of 50, the size of the complexes was 116.30±8.6 nm, and the zeta potential was 30.7±2.0 mV.

### MTT cytotoxicity assays

**Figure 2:**
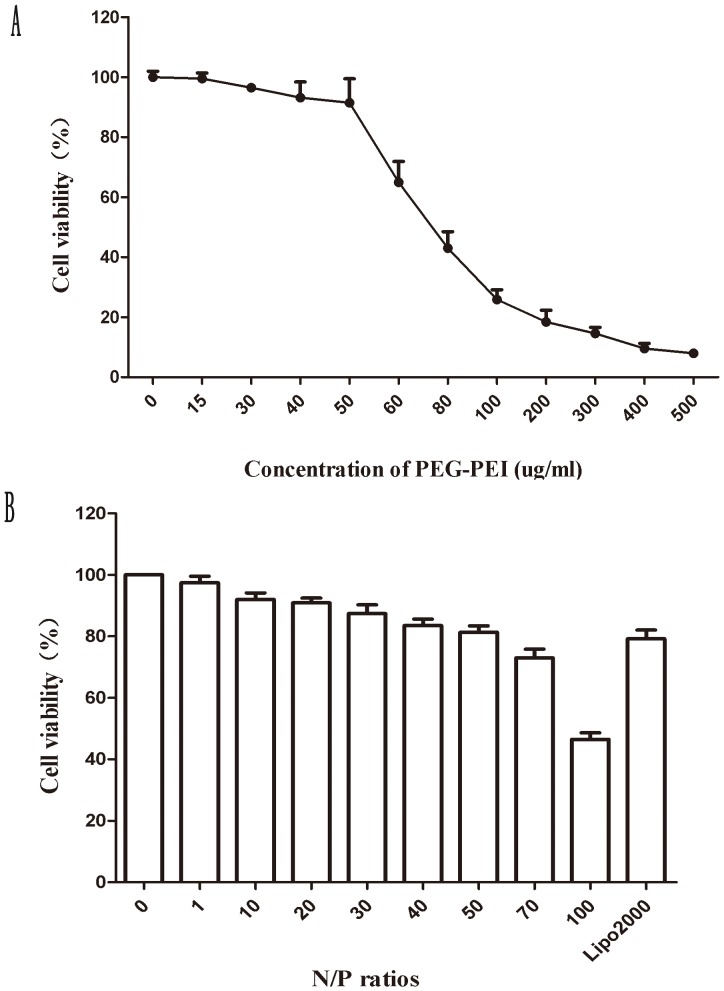
*In vitro* cytotoxicity of PEG-PEI and siROCK2 complexes toward U251 cells, as determined by an MTT assay. **A** *In vitro* cytotoxicity of PEG-PEI (at concentrations from 0 to 500 ug/ml) toward U251 cells. **B** *In vitro* cytotoxicity of siROCK2 complexes formed at different N/P ratios toward U251 cells. The concentration of siROCK2 was 1.33 ug/mL (100 nM) in each well and the incubation time was 24 h. The data are shown as the means ± SD; n = 3.

The cytotoxicity of the PEG-PEI and siROCK2 complexes was assessed by MTT assay. As shown in Fig. 2A, the cytotoxicity of PEG-PEI increased with an increase in its concentration. When the concentration of PEG-PEI reached 50 μg/mL, the cell viability was 87.97±9.56%. In addition, the cell viability was markedly decreased when the concentration was increased to an amount higher than 50 μg/mL (p<0.05). Fig. 2B demonstrates that the cytotoxicity of siROCK2 complexes increased with increasing N/P ratio. In addition, the cytotoxicity was lower than that of Lipo2000/siROCK2 at an N/P ratio of 50 (P = 0.48).

### Transfection efficiency test and cell uptake of siROCK2 complexes

**Figure 3:**
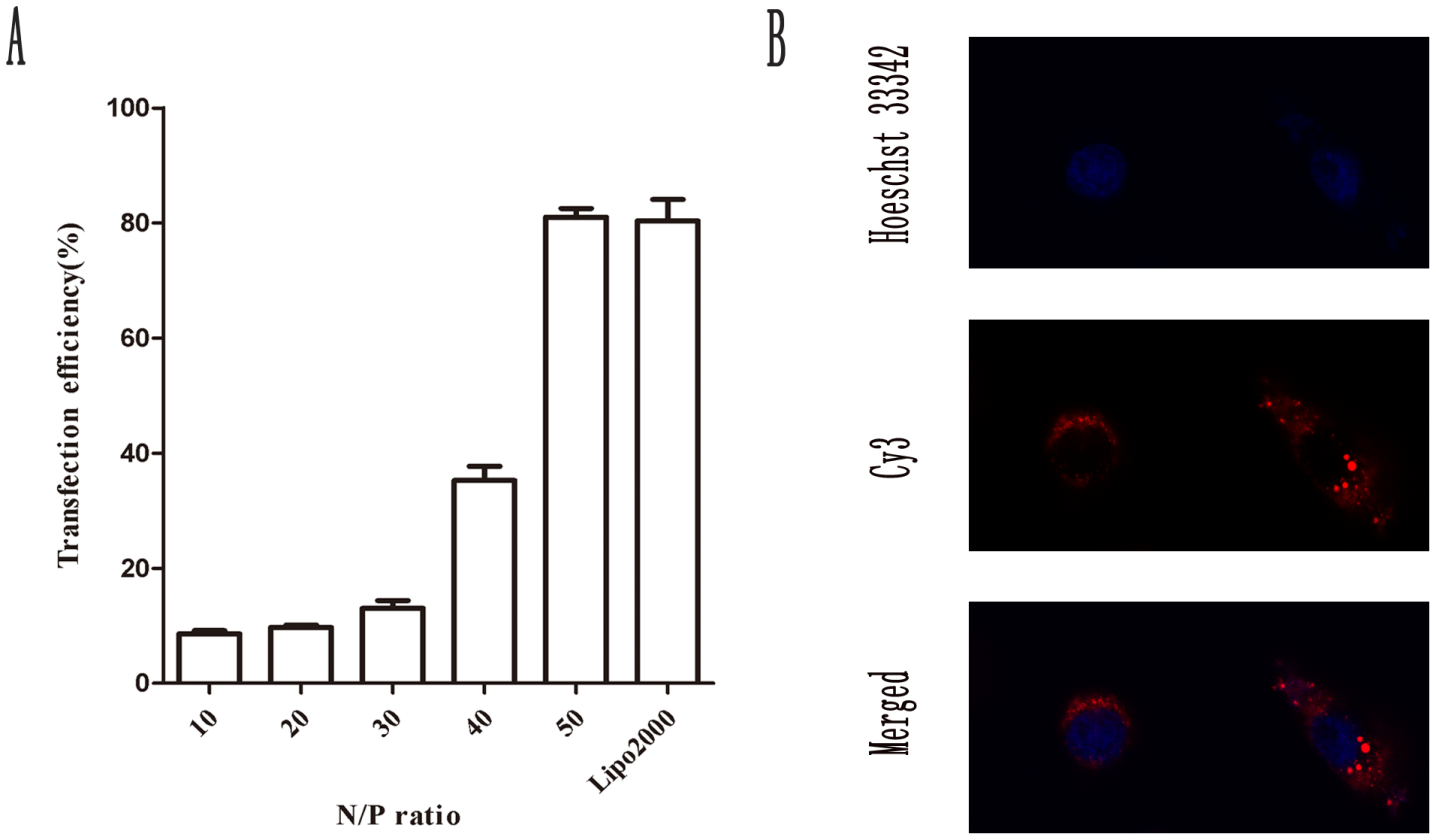
Transfection efficacy and cell uptake of siROCK2 complexes in U251 cells. **A** The ratio of Cy3-positive cells was detected by flow cytometry at various N/P ratios to analyze the transfection efficiency of siROCK2 complexes. The incubation time was 6 h. The concentration of siROCK2 was 100 nM. Lipo2000 was used as the positive control. **B** Representative LSCM images of U251 cells incubated with siROCK2 complexes at an N/P ratio of 50. The incubation time was 6 h. The concentration of siROCK2 was 100 nM. The nuclei were stained blue with Hoechst 33342, and the red fluorescence denotes Cy3-labeled siROCK2. The data are shown as the means ± SD; n = 3.

The transfection efficiency of PEG-PEI and Cy3-labeled siROCK2 complexes in U251 cells was evaluated. As shown in Fig. 3A, the transfection efficiency relied closely on the N/P ratios and increased continuously with an increase in the N/P ratio. For N/P ratios between 10 and 50, the transfection efficiencies increased from 8.61±1.16% to 81.04±1.47%. Taking into consideration both the cytotoxity and the transfection efficiency of the siROCK2 complex, we choose an N/P ration of 50 for the subsequent studies. As shown in Fig. 3B, at an N/P ratio of 50, the CLSM test demonstrated that the Cy3-labeled siROCK2 molecules were successfully uptaken and distributed within the cytoplasm, particularly in the perinuclear area.

### Downregulation of ROCK2 mRNA and protein levels

**Figure 4:**
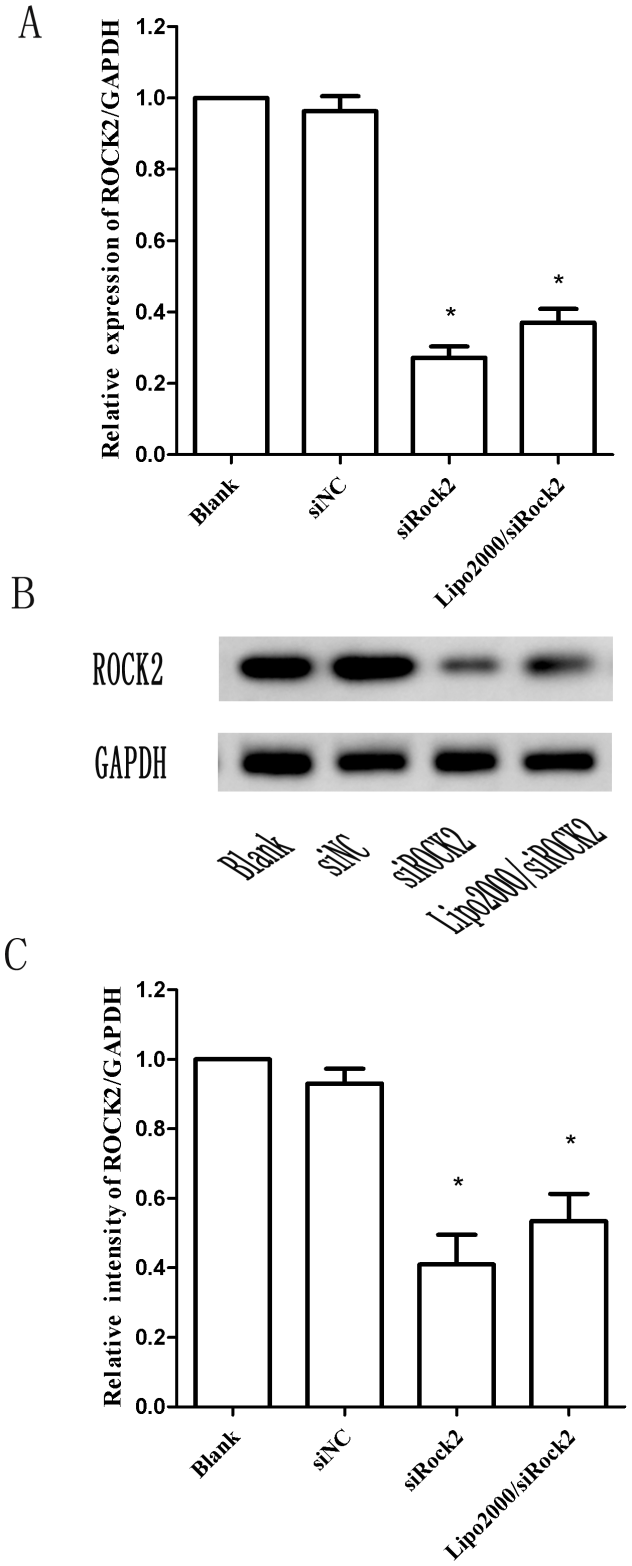
Gene silencing effects of siROCK2 complexes in U251 cells. **A** Relative mRNA expression of ROCK2 (N/P = 50) in U251 cells 24 h after transfection. **B** Suppression of ROCK2 protein expression by the siROCK2 complex (N/P  = 50) in U251 cells 48 h after transfection. **C** The relative intensity of the corresponding Western blotting bands was determined using the Image-J software. The blank control was not treated, and siNC was a negative nonsense sequence of siROCK2. Lipo2000 was used as the positive control. The concentration of siROCK2 was 100 nM. The data are shown as the means ± SD; n = 3). GAPDH was used as an internal standard. ^*^P<0.05 compared with the blank.

To validate the effects of the transfection of siROCK2 complex, we tested the mRNA and protein levels of ROCK2 24 and 48 h after transfection, respectively. As shown in Fig. 4A–B, the siROCK2 complex reduced the levels of ROCK2 mRNA and protein by 67.66±3.28% and 51.71±5.74% compared with cells transfected with siNC, respectively (P<0.05). In addition, the gene silencing effect of the siROCK2 complex was more effective compared with that of Lipo2000/siROCK2. In total, the siROCK2 complex demonstrated the effective knockdown of both ROCK2 mRNA and protein, which indicates that the siROCK2 complex is not only uptaken successfully but also functions effectively.

### Combination treatment with TMZ and siROCK2 increases apoptosis

**Figure 5:**
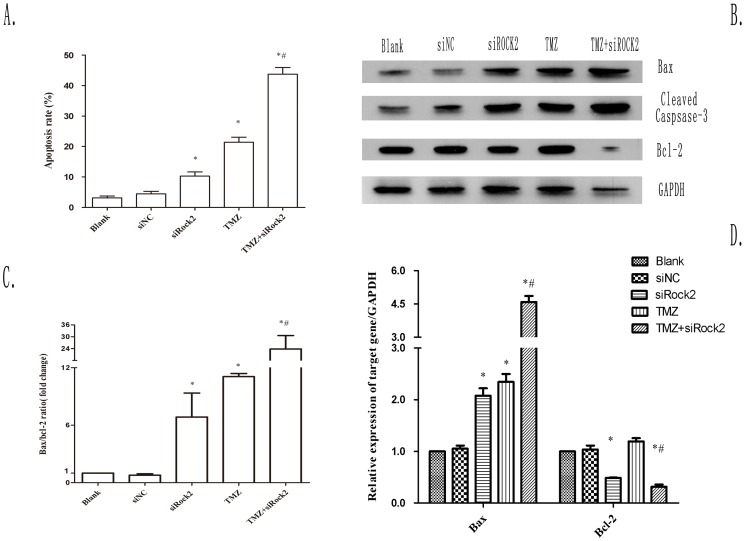
Combination treatment with TMZ and siROCK2 increases apoptosis in U251 cells. **A** The apoptosis rate was detected by flow cytometry using the Annexin V-FITC-PI double staining method. U251 cells were treated with siNC, siROCK2 complex, TMZ (100 μM), or the combination of the siROCK2 complex and TMZ (100 μM) for 48 h. **B** Representative Western blotting bands of Bax, cleaved caspase-3, and Bcl-2 in U251 cells treated as indicated. GAPDH was used as an internal standard. **C** Relative Bax/Bcl-2 ratio obtained from the corresponding Western blotting bands. **D** Relative mRNA expression of Bax and Bcl-2 in U251 cells treated with siNC, siROCK2 complex, TMZ (100 μM), or the combination of siROCK2 complex and TMZ (100 μM) for 24 h. The relative mRNA expression was determined by RT-PCR. GAPDH was used as an internal standard. The data are presented as the means ± SD; n = 3. ^*^P<0.05 compared with the blank; ^#^P<0.05 compared with siROCK2 or TMZ.

We conducted an FCM analysis to determine the apoptosis of U251 cells after various treatments for 48 h. The quantification of the data demonstrated that 21.36±1.69%, 10.26±1.36%, and 43.73±2.21% of the cells were apoptotic after treatment with TMZ, siROCK2, of their combination, respectively (Fig. 5A and Fig. S1). The results indicate that the combined treatment induced a twofold higher degree of apoptosis in U251 cells than TMZ treatment alone. We observed increases in apoptosis due to the combination treatment with TMZ and siROCK2 compared with either treatment alone. Therefore, we investigated the expression of several apoptosis-related proteins after TMZ, siROCK2 or their combination treatment in U251 cells. The Western blot analysis showed that cells treated with TMZ, siROCK2, or the combination demonstrated increased expression levels of Bax and cleaved caspase-3 and decreased expression levels of Bcl-2. However, higher fold changes were observed in the cells treated with both TMZ and siROCK2 compared with cells treated with either treatment alone (Fig. 5B and Fig. S2). It is well known that the Bax/Bcl-2 ratio plays an essential role during apoptosis. Therefore, the quantification of the Bax/Bcl-2 ratio from the corresponding Western blotting bands was conducted. As indicated in Fig. 5C, the combined treatment showed 3.51 and 2.05 fold higher Bax/Bcl-2 ratios than those obtained with TMZ or siROCK2 alone, respectively. We further examined the mRNA levels of Bax and Bcl-2 using RT-PCR, and similar results were obtained, as presented in Fig. 5D and Fig.S3 A–B.

### Combination treatment with TMZ and siROCK2 inhibits cell migration

**Figure 6:**
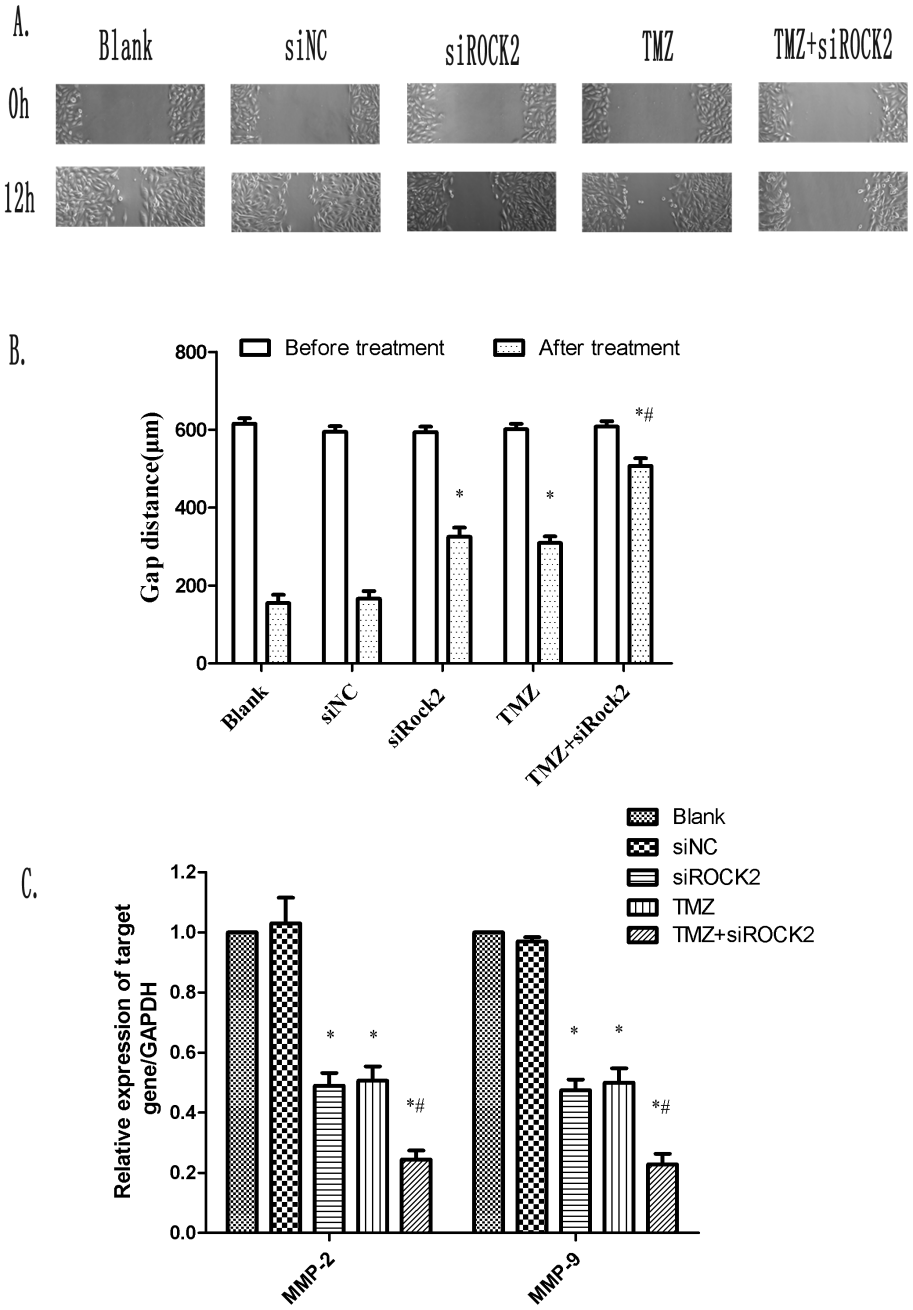
Combination treatment with TMZ and siROCK2 prevents cell migration in the wound-healing assay. **A** The images were captured using a phase contrast microscope at 0 and 12(100 μM), or the combination of siROCK2 complex and TMZ (100 μM) for 12 h. **B** The gap distance was measured using the ImageJ software and is presented in the bar graph. **C** Measurement of the mRNA levels of MMP-2 and MMP-9 after treatment for 12 h. The data are presented as the means ± SD; n = 3. ^*^P<0.05 compared with the blank; ^#^P<0.05 compared with siROCK2 or TMZ.

To examine the effect of the combination treatment of TMZ and siROCK2 on the migration of U251 cells, we conducted a wound-healing assay. Cell migration was clearly inhibited by TMZ, siROCK2, and their combination, as shown in Fig. 6A–B. After wounding the cell monolayer, the gap distance was 612.51±13.56 μm at 0 h and decreased to 166.07±19.78 μm after 12 h of incubation under the control conditions, indicating cell migration. The gap distances after 12 h of TMZ and siROCK2 treatment alone were 309.18±17.21 μm and 325.01±23.54 μm, respectively, indicating the partial inhibition of cell migration by these two individual treatments. The gap distance after the combination treatment with TMZ and siROCK2 was 505.01±20.07 μm, which is equal to 83.39% of the control distance at 0 h and thus indicates that cell migration was almost completely inhibited. These results suggest that TMZ or siROCK2 treatment alone is able to partially inhibit cell migration and that the combination treatment has an additive inhibitory effect on cell migration. Based on the wounding assay results, we investigated the effect of TMZ and siROCK2 on invasion-related gene expression using RT-PCR. The mRNA levels of MMP-2 and MMP-9 in the combination-treated cells were statistically downregulated compared with the levels observed in the siNC cells. Fig.6C and Fig.S3 C–D demonstrate the significantly downregulated levels of MMP-2 and MMP-9 in the combination-treated cells compared with those obtained in the cells treated with either treatment alone, and these results suggest a possible mechanism for the decreased cell migration.

## Discussion

An increasing body of evidence indicates that an abnormal activation of the Rho/ROCK pathway is observed in various CNS diseases, including glioma. However, the combined antitumor effect of siROCK2 and TMZ in glioma has not been well established. The key findings of the current study were the following: (i) PEG-PEI serves as a siRNA delivery system that presents substantial binding affinity for siROCK2 and exhibits low cytotoxicity in U251 cells and (ii) ROCK2 silencing through the siROCK2 complex enhances the cytotoxic effect of TMZ in U251 cells.

Among the non-viral vectors for siRNA, PEI is commonly used and offers the highest gene delivery efficiency among the cationic polymers [15]. However, PEI was chemically modified through the addition of PEG molecules to enhance its delivery efficiency and reduce its cytotoxicity. Therefore, PEG-PEI/siROCK2 complexes were synthesized, and their physical characteristics were analyzed.

The physical characteristics of the siROCK2 complex suggest that the formation of the nanoparticles is closely related to the N/P ratio. Agarose electrophoresis showed that siROCK2 is completely condensed by PEG-PEI at N/P ratios higher than 20, indicating that the working N/P ratio should be above 20. In addition, because of the electrostatic interactions between PEG-PEI and siROCK2, the particle size deceased, and the zeta potential increased with increasing N/P ratio. As shown in Fig.1, the particle size of the siROCK2 complex decreased from 242.1±6.5 nm (N/P  = 10) to 116.3±8.6 nm (N/P  = 50), which is similar to the results of our previous study [16]. Therefore, relatively higher N/P ratios result in better cellular uptake. Unfortunately, higher N/P ratios of the siROCK2 complex resulted in serious cytotoxicity to U251 cells. As indicated by the MTT results obtained using the siROCK2 complexes, N/P ratios higher than 50 reduced the cell viability below 80% and are thus not suitable for further investigation. Moreover, as shown in Fig. 3A, the PEG-PEI-mediated transfection efficiency increased with increasing N/P ratios. At an N/P ratio of 50, the transfection efficiency was 81.04±1.47%, which is slightly higher than obtained with Lipo2000/siROCK2 (80.38±3.71%). Taking into consideration the high transfection efficiency and the low cytotoxicity, an N/P ratio of 50 was chosen for the transfection of the siROCK2 complex into U251 cells. The CLSM test showed that the siROCK2 (N/P  = 50) complexes successfully crossed the cytomembrane because these were localized in the cytoplasm, which further confirmed that an N/P ratio of 50 is the optimal choice for the transfection of the siROCK2 complex into U251 cells. The downregulation of the protein and mRNA levels of ROCK2 demonstrates that the siROCK2 complex functioned correctly after transfection.

It is well known that improved chemotherapeutics are essential for better prognosis of patients with malignant glioma, but chemoresistance is a hallmark of glioma. Our results demonstrate that a combination treatment with TMZ and siROCK2 induces increased apoptosis of U251 cells than either treatment alone, indicating the siROCK2 exerts a sensitization effect on TMZ. Although the cytotoxicity of a ROCK2 inhibitor, namely Fasudil, was previously investigated in glioma cells, to the best of our knowledge, this study provides the first demonstration of the synergistic antitumor effects of siROCK2 with TMZ.

Apoptosis is regulated by some protein families, including the Bcl-2 family and the caspase family [17]. Previous studies have shown that glioma cells treated with TMZ exhibit changes in the expression levels of Bax and Bcl-2, which are involved in the mitochondrial pathway of apoptosis [18]. Thus, we further investigated the expression levels of Bax and Bcl-2 in U251 cells after the combined treatment by Western bolting and RT-PCR. The results demonstrate that the cells treated with the combined treatment of siROCK2 and TMZ appeared to exhibit an increased level of Bax and a decreased level of Bcl-2, which indicates that siROCK2 has a sensitization effect on TMZ. In addition, the increased Bax/Bcl-2 ratios observed in the combination-treated cells were higher than those observed in cells treated with either individual treatment. Therefore, we concluded that the proapoptotic effect of the TMZ and siROCK2 combination may be achieved through the enhancement of the Bax/Bcl-2 ratio.

Because caspases-3 is a critical mediator of the mitochondrial events of apoptosis [19], the cleaved caspase-3 activity was measured by Western blotting. As shown in Fig. 5A, the levels of cleaved caspase-3 were slightly increased in response to siROCK2 or TMZ treatment compared with the blank control or siNC group. However, these effects were enhanced about two or three fold as a result of the combination treatment with TMZ and siROCK2 than either alone (Fig. S2). These results indicate that siROCK2 could, at least in part, increase the activation of the mitochondria-related apoptosis induced by TMZ.

One of the hallmarks of human malignant glioma cells is their highly invasive nature. Our results indicate that the inhibition of siROCK2 significantly suppresses the invasiveness compared with the control U251 cells, as determined through the wound-healing assay. In addition, the gap distance obtained after the combination treatment with TMZ and siROCK2 was significantly reduced compared with that obtained with either treatment alone, suggesting that siROCK2 has a sensitizing effect on TMZ during the inhibition of cell migration.

Strong correlations have been reported between the overexpression of MMPs and enhanced tumor invasiveness in human glioma. A previous study demonstrated that the downregulation of MMP-9 expression in glioma cells significantly decreases their migration and invasiveness [20]. Our results indicate that the inhibition of siROCK2 significantly suppresses the expression of MMP-2 and MMP-9. The results show some differences with those of a previous study, which showed that Fasudil exerts a dose-dependent inhibition effect on MMP-2 expression but not MMP-9 expression in GBM cells [9]. Interestingly, the opposite results demonstrated that the knockdown of ROCK2 leads to a significant increase in the MMP-9 and MMP-2 levels in IEC-6 cells [21]. We do not know the reason for this difference but hypothesize that ROCK2 may participate in different pathways to regulate these MMPs in different tumors, and further investigation is needed to elucidate the underlying mechanisms.

In addition, the TMZ-treated cells also demonstrated the downregulated expression of MMP-2 and MMP-9, which is in agreement with the enhanced gap distance obtained in the wound-healing assay. Although other studies have shown no influence or an increase in the expression level of MMP-2 and MMP-9 in TMZ-treated cells, the different results most likely derive from the different cell lines or model systems used in the different studies [22], [23]. As shown in Fig. 6C, the combination treatment with TMZ and siROCK2 significantly reduced the expression of MMP-2 and MMP-9 than compared with either treatment alone, suggesting a possible mechanism for the decreased cell migration.

In conclusion, we found that PEG-PEI served as a siRNA delivery system that presents substantial binding affinity for siROCK2 and exhibits low cytotoxicity toward U251 cells. Moreover, the downregulation of ROCK2 combined with TMZ treatment exerted synergistic effects on the induction of apoptosis and the inhibition of cell migration in U251 glioma cells.

## Supporting Information

Figure S1 **Representive dot plots of the results of the apoptosis assay and the percentage of cells in each quadrant.** a blank group; b siNC group; c siROCK2 group; d TMZ group; e TMZ+ siROCK2 group;
(TIF)

Figure S2 **The quantification of the corresponding western blotting bands using the ImageJ software.** The data are presented as the (Means ± SD; n = 3). ^*^P<0.05 compared with the blank; ^#^P<0.05 compared with siROCK2 or TMZ.
(TIF)

Figure S3 **The quantification of q-PCR assay (Bax, Bcl-2, MMP-2 and MMP-9) using the 2^−ΔΔCt^ method.** The data are presented as the (Means ± SD; n = 3). ^*^P<0.05 compared with TMZ +siROCK2.
(TIF)

## References

[1] Kohler BA, Ward E, McCarthy BJ, Schymura MJ, Ries LA, et al (2011) Annual report to the nation on the status of cancer, 1975–2007, featuring tumors of the brain and other nervous system. J Natl Cancer Inst 103: 714–736. 21454908 10.1093/jnci/djr077 PMC3086878

[2] Stupp R, Mason WP, van den Bent MJ, Weller M, Fisher B, et al (2005) Radiotherapy plus concomitant and adjuvant temozolomide for glioblastoma. N Engl J Med 352: 987–996. 15758009 10.1056/NEJMoa043330

[3] Schofield AV, Bernard O (2013) Rho-associated coiled-coil kinase (ROCK) signaling and disease. Crit Rev Biochem Mol Biol 48: 301–16. 23601011 10.3109/10409238.2013.786671

[4] Riento K, Ridley AJ (2003) Rocks: multifunctional kinases in cell behaviour. Nat Rev Mol Cell Biol 4: 446–456. 12778124 10.1038/nrm1128

[5] Zohrabian VM, Forzani B, Chau Z, Murali R, Jhanwar-Uniyal M (2009) Rho/ROCK and MAPK signaling pathways are involved in glioblastoma cell migration and proliferation. Anticancer Res 29: 119–123. 19331140

[6] Yan B, Chour HH, Peh BK, Lim C, Salto-Tellez M (2006) RhoA protein expression correlates positively with degree of malignancy in astrocytomas. Neurosci Lett 407: 124–126. 16978776 10.1016/j.neulet.2006.08.032

[7] Erkutlu I, Cigiloglu A, Kalender ME, Alptekin M, Demiryurek AT, et al (2013) Correlation between Rho-kinase pathway gene expressions and development and progression of glioblastoma multiforme. Tumour Biol 34: 1139–1144. 23338717 10.1007/s13277-013-0655-9

[8] Oellers P, Schroer U, Senner V, Paulus W, Thanos S (2009) ROCKs are expressed in brain tumors and are required for glioma-cell migration on myelinated axons. Glia 57: 499–509. 18814230 10.1002/glia.20777

[9] Deng L, Li G, Li R, Liu Q, He Q, et al (2010) Rho-kinase inhibitor, fasudil, suppresses glioblastoma cell line progression in vitro and in vivo. Cancer Biol Ther 9: 875–884. 20364104 10.4161/cbt.9.11.11634

[10] Liang Y, Liu Z, Shuai X, Wang W, Liu J, et al (2012) Delivery of cationic polymer-siRNA nanoparticles for gene therapies in neural regeneration. Biochem Biophys Res Commun 421: 690–695. 22542938 10.1016/j.bbrc.2012.03.155

[11] Wang J, Lei Y, Xie C, Lu W, Yan Z, et al (2013) Targeted gene delivery to glioblastoma using a C-end rule RGERPPR peptide-functionalised polyethylenimine complex. Int J Pharm 458: 48–56. 24144951 10.1016/j.ijpharm.2013.10.017

[12] Neu M, Germershaus O, Behe M, Kissel T (2007) Bioreversibly crosslinked polyplexes of PEI and high molecular weight PEG show extended circulation times in vivo. J Control Release 124: 69–80. 17897749 10.1016/j.jconrel.2007.08.009

[13] Alexander S, Friedl P (2012) Cancer invasion and resistance: interconnected processes of disease progression and therapy failure. Trends Mol Med 18: 13–26. 22177734 10.1016/j.molmed.2011.11.003

[14] Zhang S, Wan Y, Pan T, Gu X, Qian C, et al (2012) MicroRNA-21 inhibitor sensitizes human glioblastoma U251 stem cells to chemotherapeutic drug temozolomide. J Mol Neurosci 47: 346–356. 22528454 10.1007/s12031-012-9759-8

[15] Godbey WT, Wu KK, Mikos AG (1999) Poly (ethylenimine) and its role in gene delivery. J Control Release 60: 149–160. 10425321 10.1016/s0168-3659(99)00090-5

[16] Liu Y, Liu Z, Wang Y, Liang YR, Wen X, et al (2013) Investigation of the performance of PEG-PEI/ROCK-II-siRNA complexes for Alzheimer's disease in vitro. Brain Res 1490: 43–51. 23103413 10.1016/j.brainres.2012.10.039

[17] Gross A, McDonnell JM, Korsmeyer SJ (1999) BCL-2 family members and the mitochondria in apoptosis. Genes Dev 13: 1899–1911. 10444588 10.1101/gad.13.15.1899

[18] Das A, Banik NL, Patel SJ, Ray SK (2004) Dexamethasone protected human glioblastoma U87MG cells from temozolomide induced apoptosis by maintaining Bax: Bcl-2 ratio and preventing proteolytic activities. Mol Cancer 3: 36. 15588281 10.1186/1476-4598-3-36 PMC544397

[19] Lakhani SA, Masud A, Kuida K, Porter GJ, Booth CJ, et al (2006) Caspases 3 and 7: key mediators of mitochondrial events of apoptosis. Science 311: 847–851. 16469926 10.1126/science.1115035 PMC3738210

[20] Kondraganti S, Mohanam S, Chintala SK, Kin Y, Jasti SL, et al (2000) Selective suppression of matrix metalloproteinase-9 in human glioblastoma cells by antisense gene transfer impairs glioblastoma cell invasion. Cancer Res 60: 6851–6855. 11156378

[21] Vishnubhotla R, Sun S, Huq J, Bulic M, Ramesh A, et al (2007) ROCK-II mediates colon cancer invasion via regulation of MMP-2 and MMP-13 at the site of invadopodia as revealed by multiphoton imaging. Lab Invest 87: 1149–1158. 17876296 10.1038/labinvest.3700674

[22] Trog D, Yeghiazaryan K, Fountoulakis M, Friedlein A, Moenkemann H, et al (2006) Pro-invasive gene regulating effect of irradiation and combined temozolomide-radiation treatment on surviving human malignant glioma cells. Eur J Pharmacol 542: 8–15. 16806166 10.1016/j.ejphar.2006.05.026

[23] Gabelloni P, Da PE, Bendinelli S, Costa B, Nuti E, et al (2010) Inhibition of metalloproteinases derived from tumours: new insights in the treatment of human glioblastoma. Neuroscience 168: 514–522. 20382206 10.1016/j.neuroscience.2010.03.064

