# Correction: Downregulation of ROCK2 through Nanocomplex Sensitizes the Cytotoxic Effect of Temozolomide in U251 Glioma Cells

**DOI:** 10.1371/journal.pone.0101149

**Published:** 2014-06-18

**Authors:** The PLOS ONE Staff

There are errors in the corresponding author designation. Zhonglin Liu should not be noted as contributing equally to this work, but rather is the corresponding author (zhonglinliu@126.com).
