# Supplementary figures and images for: Downregulation of ROCK2 through Nanocomplex Sensitizes the Cytotoxic Effect of Temozolomide in U251 Glioma Cells
Source: PLoS One. 2014 Mar 18;9(3):e92050. doi: 10.1371/journal.pone.0092050 (PMC3958422; doi:10.1371/journal.pone.0092050)

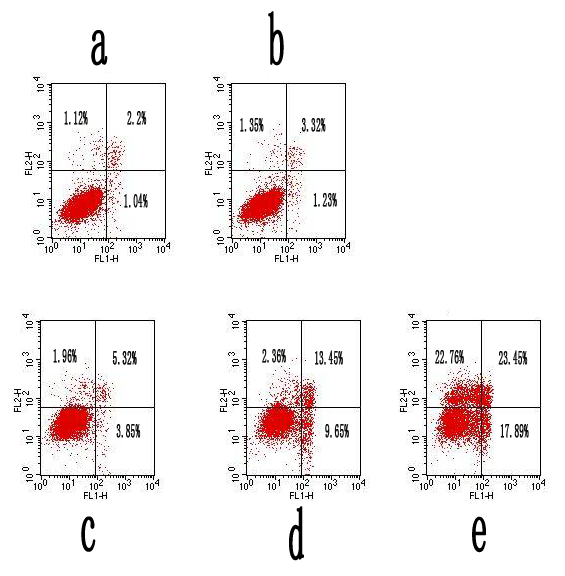

Supplement: Figure S1 — Representive dot plots of the results of the apoptosis assay and the percentage of cells in each quadrant. a blank group; b siNC group; c siROCK2 group; d TMZ group; e TMZ+ siROCK2 group; (TIF) [file pone.0092050.s001.tif]

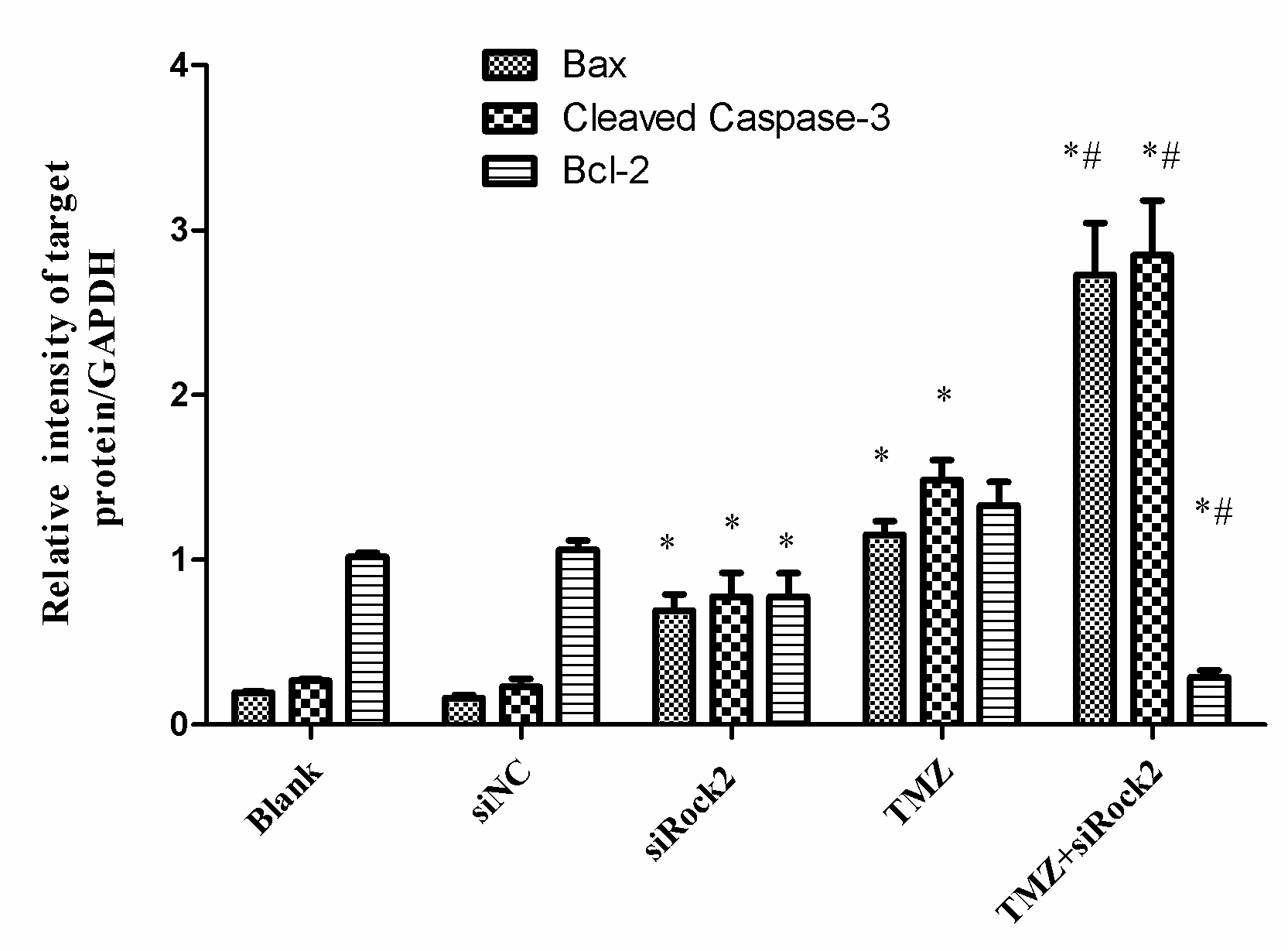

Supplement: Figure S2 — The quantification of the corresponding western blotting bands using the ImageJ software. The data are presented as the (Means ± SD; n = 3). *P<0.05 compared with the blank; #P<0.05 compared with siROCK2 or TMZ. (TIF) [file pone.0092050.s002.tif]

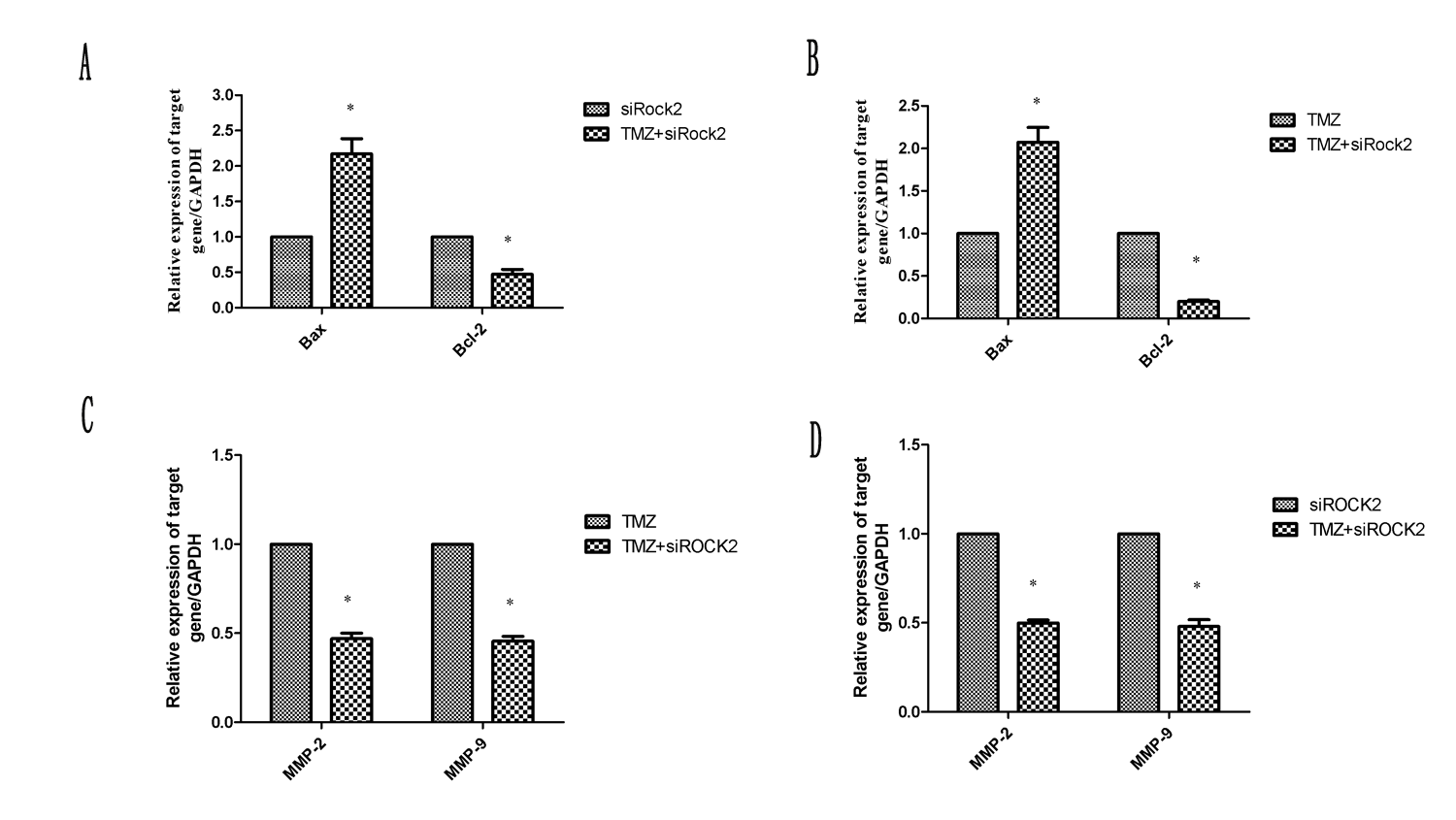

Supplement: Figure S3 — The quantification of q-PCR assay (Bax, Bcl-2, MMP-2 and MMP-9) using the 2−ΔΔCt method. The data are presented as the (Means ± SD; n = 3). *P<0.05 compared with TMZ +siROCK2. (TIF) [file pone.0092050.s003.tif]
